# On the Manna of the Hebrews

**Published:** 1860-10

**Authors:** M. O'Rorke


### ARTICLE XVIII.

### *On the Manna of the Hebrews.* By M. O'Rorke.

All commentators upon the Scriptures have, until now, regarded the substance upon which the Israelites were nourished in the wilderness for forty years, as a true manna. From the recent investigations of Dr. O'Rorke it becomes evident that this manna was a kind of lichen, and, very probably, analogous to a species which he has shown in Paris, which was derived from Algeria.

The following is an abstract of Dr. O'Rorke's communication :—We read in the 16th chapter of the Book of Exodus, that "The Israelites took their journey from Elim, and came into the wilderness of Sin. God came to the succor of his people. At even the quails came up and covered the camp ; and in the morning a thick dew lay round about. It was a kind of white grain suitable for making bread. It had the taste of the purest flour mixed with milk. When the children of Israel saw it, they said one to another *Manhu!* What is this ?" From this exclamation arose the word manna. "It covered the camp at the dawn of day, and melted at the first rays of the sun. This manna, when pounded, could be formed into a paste, and baked as bread, or made up in several ways in pastry."

The Israelites speedily took a dislike to this food, for a little further on (Numbers, chap. xi., v. 6-8) we read again: "But now our soul is dried away, there is nothing at all besides this manna before our eyes, and the manna was as coriander seed, and the color thereof as the color of bdellium." The people went about in quest of it, and having gathered it they ground it in mills, or beat it in a mortar, and baked it in pans, and made cakes of it, which had the taste of unleavened bread kneaded with oil. Dr. O'Rorke has remarked that the various translators of the Hebrew text differ amongst themselves as to the taste of the manna. One version of the 31st verse of the 16th chapter of Exodus is thus rendered: This manna was like coriander seed, it was white, and it had a taste resembling the purest flour mixed with honey. The translators have evidently confounded the expression sweet, little sapid, insipid, and sugary, since they refer the flavor of manna to that of milk, of honey, and even of oil. Now, in all the European languages the word manna is employed to designate a kind of sugary gum,—a concrete sap, which exudes from certain trees in Sicily, Calabria, and Spain, either spontaneously, or by the puncture of insects, or by artificial incisions. The officinal manna is obtained from a species of ash (*fraxinus ornus* and *rotundifolia*,) and is collected in June and July. The Sicilian manna is purgative, and it is clear that this manna is not that of the Hebrews. But there are many other kinds of manna known; it is certain even, that this name has been extended to some sugary or resinous substances, and to true honeys. In Europe the larch (*larix europœa*) yields a concrete juice called manna of Briançon or manna of the larch. The juniper also, and in Sweden the *pinus picea* yield some at the extremities of their highest branches. The East also furnishes several kinds of manna derived from different plants, in Syria, Persia and Arabia. These are obtained from the cedar, from a leguminous plant (*hedysarum alhagi*,) from the oriental oak, (?) from the tamarisk, and from several other undetermined species of plants.

All these mannas are collected upon the branches and the leaves of the trees which produce them, or upon the ground beneath the same trees. They assume the form of little seeds like to coriander, sometimes as white as snow, which the inhabitants collect in June and August before the sun rises; for when the sun has risen, these little masses melt, and form a honey-colored coating which does not separate spontaneously from the branches. Rauwolf, Gmelin, Niebuhr, and especially Burckardt, have given very correct descriptions of these mannas, and of the plants which produce them.

Burckardt, in the account of his travels in the deserts of Sinai, which were traversed by the Israelites, mentions the tarfa or tamarisk as being the plant which furnishes the manna of the Hebrews. It is really strange, says he, that this should have remained unknown in Europe until it was indicated by M. Seetzen. This substance is called mann by the Arabs, and resembles, up to a certain point, the description of manna given in the Scriptures. It may be remarked further, that this manna is only found in very wet seasons; sometimes it is altogether wanting. In the season during which the Arabs collect it, it never acquires that degree of firmness which will allow of its pulverization. The amount of tamarisk manna really collected, even in the most favorable seasons, is insignificant, and does not exceed five hundred livres for the whole of the country. It is entirely consumed by the Bedouins, who regard it as the most delicate dainty which the country furnishes. The cedars of Lebanon also yield a manna which resembles that of the larch, called cedrine, mastichina; in Egypt, the *asclepias procera* also produces a farinaceous manna, and in Syria the *apocynum syriacum* is in the same estimation. But all these mannas, and especially that of the tamarisk, differ very much from the manna of the Israelites. The manna of the Hebrews fell from heaven, and covered the camp on the ground; whilst the real oriental mannas are obtained from the stems, branches, or leaves of the trees from which they exude, and cannot be dispersed into the atmosphere and then fall in the form of dew. It is true that, anciently, all the mannas were designated by the expressions of dew of heaven, honey of the air, heavenly honey, because the ancients imagined that the sugary drops suspended from the trees were produced by the dew which hardened upon those plants. Such was the opinion of Aristotle, Pliny, and Avicenna. Mathiolus regarded it as a sort of saliva or excrement from certain stars; and it was not till 1843 that all these absurd beliefs were shown to be wrong by Angus Palea, who demonstrated that Sicilian manna did not fall from heaven in the form of dew; he proved that it was produced directly from the proper juice of the ash, by simply covering such trees with a white cloth, and thus isolating them from external influences. Moreover, the supply of Eastern manna often fails for one or more years in succession; it is always anything but abundant, the trees only producing it for two or three months in the year. The manna of the Bible, on the contrary, fell all the year, and continued to do so for the long period of forty years, without intermission. The real manna is only employed as a condiment or dainty—that of the Hebrews served for their daily bread. Moreover, until now, no travelers have related that the Arabs really use manna to replace their bread during meals.

The manna of the Hebrews was not, then, a true manna, and Dr. O'Rorke believes that he can demonstrate evidently that this manna was a *lichen*. The lichens are cryptogamous, and there are a great many species. They are the first plants which make their appearance upon naked bodies, such as stones, rocks and the ground. These plants have no true roots, and are not parasitic, except in appearance, for they do not live at the expense of the body upon which they are applied and to which they adhere. Heat dries them up, but moisture restores them to life. No lichen is deleterious, and all contain a nitrogenous matter, and in abundance a kind of starch; they are employed, therefore, in several parts of the globe as food for man and other animals. We could even employ them everywhere for such a purpose, did we not possess more nourishing matters, and of more agreeable flavor. In Lapland the reindeer eat them; in Norway, those of the inhabitants which are nourished by them are said to be less frequently affected by leprosy than those of whom fish constitutes the principal food. The alimentary value of certain lichens would be even superior, according to some authors, to that of wheat, since a bushel of powdered lichen is equivalent to two bushels of wheat-flour. A great many lichens contain enormous quantities of oxalate of lime. Some contain a bitter principle (*cetrarine*, &c.,) which renders them febrifugal ; others a rich coloring principle (*orcine* :) and all contain a fecula analogous to inuline, which is not colored blue by iodine.

Amongst the lichens, there is one which is called by Pallas, lichen esculentus, and which, according to Acharius, belongs to the genera lecanora and (?) parmelia. This lichen is found in Persia, in the deserts of Tartary, in the Crimea, in Asia Minor, &c., always on the ground, where it is carried either by the winds or by its falling from the neighboring mountains. It there sometimes forms beds several inches in thickness. The sheep are nourished by it, and men make of it a kind of bread, which the poor consume and regard as true manna sent to them by Providence. Already, on the 3d of August, 1828, Thénard had presented to the Academy of Sciences a lichen of a fawn color, granulated, composed of broken crusts, which had fallen in the neighborhood of Mount Ararat, in Armenia, and which a Russian general of the Persian army had sent to him.

It appears that this lichen dries up during the summer upon the mountains, and is transported by the winds to a great distance, which has caused the inhabitants to say that this grain fell from heaven. This shower was not rare, and under certain circumstances covered the ground to a depth of five or six inches in several places. The sheep were very fond of it, and men were habitually nourished by it. A shower of this kind was noticed in 1845 in the Crimea, at Jenis-Bechir. It covered the ground to a depth of three or four inches, and the inhabitants, following the example of Dr. Leveillé, nourished themselves with it for several days. This lichen is even more common in the Algerian Sahara, and in Arabia. Everywhere they employ it for the nourishment of men and horses. The specimen lately shown in Paris by Dr. O'Rorke was collected by M. Bellesterot at Boghar, in Algeria, and M. Hardy, Director of the Botanic Gardens of Algiers in 1849, had sent a specimen of it to the Exposition, which was altogether unnoticed. This lichen called *takaout* in Arabia, and *ousseh elard* (excrement of the earth) in Algeria, is found in the form of little twisted rounded grains, the largest of the size of a pea, and of a yellowish-grey earthy color. Its fractured surface is white and farinaceous, and contains some particles of sand which crack under the teeth. In flavor it is insipid, amylaceous, and with a feeble aroma of the champignon. When boiled in water this lichen slightly swells, becomes more tender, and can be mixed with milk, butter and salt, and form a food which has no bad quality or disagreeable appearance.

In the Algerian Sahara, as well as in Arabia, this lichen does not adhere to any foreign body; it appears to spring spontaneously from the ground after rain ; the wind collects it together in certain places in large heaps, and they say of it *acquirit vires eundo*, for in its wandering course it vegetates and increases in size. Its surface is covered with small, very apparent fructifications, from which, at maturity, the reproductive sporidia escape, which, microscopic in appearance, become dispersed over the ground, or are transported by the winds to enormous distances, in order to develop themselves, when the conditions of soil and humidity are favorable to them. Such, while it remains, is of an inestimable value to the wandering and migratory tribes of the deserts, who are preserved by it from hunger in years of famine and in certain particularly crtical circumstances.

Is it not evident, says Dr. O'Rorke, that this substance is the true manna of the Hebrews,—that which fed them with regularity for forty years in the wilderness? Those who desire a more complete agreement with the text of the Scriptures can yet admit this conclusion:—Moses has confounded under the name of manna, two distinct substances, because they both resemble each other in apparently falling from heaven, that is, 1st. An amylaceous substance which could be preserved and pulverized, suitable for making bread, might be collected at any time, increasing on the ground, like to coriander or bdellium in color; this is the lichen described above. 2nd. A sugary substance, very readily alterable, somewhat clear, and collected on certain trees or shrubs during three months of the year only, and serving as a condiment or dainty to mix with the lichen bread: that is to say, the manna of the tamarisk, of the Alhagi, and perhaps of some others. We see, then, that the real bread of the Hebrews, the manna of the wilderness, is no other than the *lichen esculentus* of Pallas, or the *lecanora esculenta* of Acharius. No commentator has hitherto alluded to this.—*Jour de Phar. et de Chimie*, and *Pharmaceutical Journal.*

